# Aging effects on intestinal homeostasis associated with expansion and dysfunction of intestinal epithelial stem cells

**DOI:** 10.18632/aging.101279

**Published:** 2017-08-29

**Authors:** Emily C. Moorefield, Sarah F. Andres, R. Eric Blue, Laurianne Van Landeghem, Amanda T. Mah, M. Agostina Santoro, Shengli Ding

**Affiliations:** ^1^ Department of Cell Biology and Physiology, University of North Carolina, Chapel Hill, NC 27599, USA; ^2^ Division of Gastroenterology, University of Pennsylvania, Philadelphia, PA 19104, USA; ^3^ Department of Molecular Biomedical Sciences, College of Veterinary Medicine, North Carolina State University, Raleigh, NC 27695, USA; ^4^ Department of Hematology, Stanford University, Stanford, CA 94305, USA; ^5^ Massachusetts General Hospital and Harvard Medical School, Boston, MA 02115, USA

**Keywords:** intestinal epithelial stem cells, aging, Sox9, organoid

## Abstract

Intestinal epithelial stem cells (IESCs) are critical to maintain intestinal epithelial function and homeostasis. We tested the hypothesis that aging promotes IESC dysfunction using old (18-22 months) and young (2-4 month) Sox9-EGFP IESC reporter mice. Different levels of Sox9-EGFP permit analyses of active IESC (Sox9-EGFP^Low^), activatable reserve IESC and enteroendocrine cells (Sox9-EGFP^High^), Sox9-EGFP^Sublow^ progenitors, and Sox9-EGFP^Negative^ differentiated lineages. Crypt-villus morphology, cellular composition and apoptosis were measured by histology. IESC function was assessed by crypt culture, and proliferation by flow cytometry and histology. Main findings were confirmed in Lgr5-EGFP and Lgr5-LacZ mice. Aging-associated gene expression changes were analyzed by Fluidigm mRNA profiling. Crypts culture from old mice yielded fewer and less complex enteroids. Histology revealed increased villus height and Paneth cells per crypt in old mice. Old mice showed increased numbers and hyperproliferation of Sox9-EGFP^Low^ IESC and Sox9-EGFP^High^ cells. Cleaved caspase-3 staining demonstrated increased apoptotic cells in crypts and villi of old mice. Gene expression profiling revealed aging-associated changes in mRNAs associated with cell cycle, oxidative stress and apoptosis specifically in IESC. These findings provide new, direct evidence for aging associated IESC dysfunction, and define potential biomarkers and targets for translational studies to assess and maintain IESC function during aging.

## INTRODUCTION

Aging is a complex process resulting in decreased tissue function, and cellular and molecular damage. Adult stem cells that self-renew and maintain progenitors that appropriately differentiate into functional cell types are essential to maintain tissue homeostasis and repair throughout lifespan [1]. Aging-associated exhaustion and dysfunction have been reported in highly prolife-rative hematopoietic stem cells (HSC) [2], hair follicle stem cells (HFSC) [3], muscle stem cells [4] and adipose-derived mesenchymal stem cells [5]. Reduced stem cell function contributes to aging associated degenerative diseases [6], and is associated with molecular changes including accumulation of reactive oxygen species (ROS) [7], defects in cell cycle regulation [8] and cellular senescence [5].

Rapid renewal of the intestinal epithelium is driven by resident intestinal epithelial stem cells (IESC). IESC divide to self-renew and generate progenitor cells that undergo rapid proliferation followed by terminal differentiation into cell types including enterocytes, Paneth cells, goblet cells and enteroendocrine cells (EEC) [9]. Differentiated cells perform absorptive, barrier, antimicrobial and secretory functions, and endocrine and metabolic regulatory functions essential to health. In *Drosophila*, aging is associated with increased IESC number and proliferation, and IESC dysfunction, including activation of stress response pathways such as JNK [10] and p38-MAPK [11]. In mammals, it has been demonstrated that aging is associated with decreased intestinal barrier function [12], impaired nutrient absorption [13], small bowel bacterial overgrowth [14] and increased risk of gastrointestinal (GI) cancers [15, 16]. Previous reports in mouse models of accelerating aging indicate phenotypic changes in the intestinal epithelium including defective regeneration, cell cycle dys-regulation [17] and altered Wnt signaling [18], indicating that aging may inhibit IESC/progenitor cell maintenance or function.

Prior studies in rodent models suggest that aging may be associated expansion of IESC or progenitor cells, but with reduced capacity for tissue repair following injury [19-22]. However, the direct effects of aging on IESC could not be examined in these studies due to a lack of IESC specific biomarkers to permit direct IESC visualization, isolation and functional analyses. The identification of specific biomarkers of IESC such as Lgr5 [23] and the development of fluorescent IESC reporter mice allow direct IESC evaluation in vivo and IESC isolation for ex vivo studies and molecular analyses [9]. Sox9-EGFP reporter mice provide a useful model because distinct levels of enhanced green fluorescent protein (EGFP) expression mark different intestinal epithelial cell (IEC) populations [24, 25]. Sox9-EGFP^High^ cells are hormone-producing EEC and cells with functional characteristics of activatable, reserve IESC [25, 26]. Sox9-EGFP^Low^ cells are active IESC also termed crypt based columnar cells (CBC) [23, 27]. Sox9-EGFP^Low^ cells have been functionally validated as IESC based on the ability of single Sox9-EGFP^Low^ cells to form enteroids in vitro when isolated from uninjured small intestine [24, 25, 28]. Sox9-EGFP^Low^ cells are enriched for Lgr5 mRNA and many other mRNAs expressed at high levels in Lgr5-expressing IESC-CBC [25, 29]. Sox9-EGFP^Sublow^ cells are rapidly dividing progenitor cells that give rise to differentiated progeny [24, 25]. Sox9-EGFPN^egative^ cells contain differentiated IEC, particularly absorptive enterocytes, Paneth cells and goblet cells [25], based on histologic analysis and high level expression of biomarkers of these cell types.

This study used Sox9-EGFP, Lgr5-EGFP and Lgr5-LacZ reporter models to test the hypothesis that aging leads to IESC dysfunction and altered intestinal epithelial homeostasis. Results reveal new and direct evidence for IESC dysfunction in old mice associated with IESC hyperproliferation and expansion but increased apoptosis. These functional changes in IESC were associated with altered expression of mRNAs encoding proteins and pathways that stimulate cell cycle progression, DNA damage and apoptosis specifically in IESC, providing new biomarkers and potential mechanisms of IESC aging.

## RESULTS

### Aging-associated decreases in enterosphere formation and enteroid development

**Figure 1:**
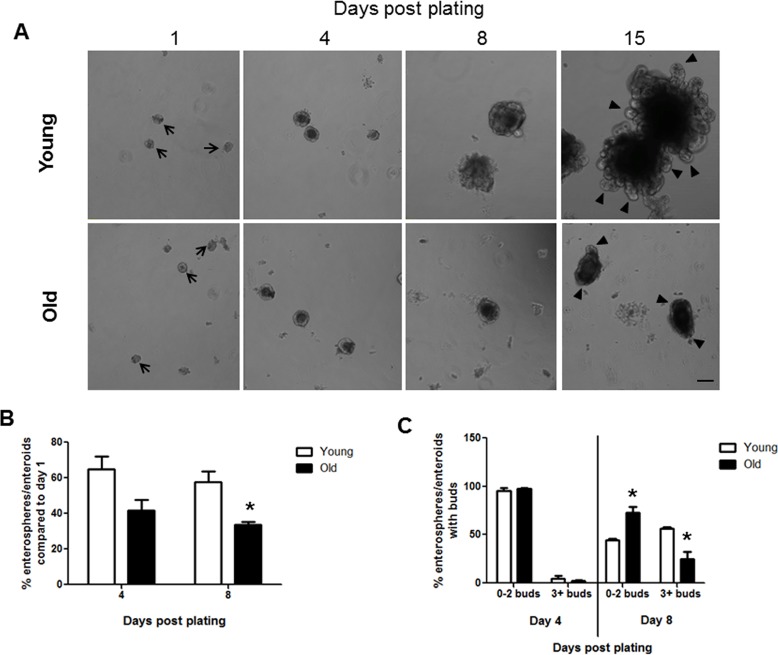
Decreased enteroid forming efficiency and budding of crypts in enteroids from old compared to young animals (**A**) Representative images of enterospheres and enteroids formed from crypts isolated from young and old mice and cultured in matrigel. Enterospheres are indicated by the black arrows. Buds are indicated by black triangles. Magnification : 10x, Scale bar : 100μm. (**B**) Quantification of enterospheres counted at day 1 that are able to grow into enteroids in matrigel culture. n=3 animals per group and 4-5 wells per animal, *p<0.05 Young vs Old, unpaired t test. (**C**) Quantification of enterosphere and enteroid complexity. n=3 animals per group and 4-5 wells per animal, *p<0.05 Young vs Old, unpaired t test.

Crypt culture in a 3D matrigel system provides a useful and widely used system to assess IESC function [30]. Isolated intestinal crypts form round enterospheres that expand and differentiate to form more complex enteroids with bud structures after several days in culture [31]. Crypt culture revealed that intestinal crypts isolated from both young and old animals formed enterospheres *in vitro* on day 1 after plating (Figure 1A). Efficiency of crypt culture was calculated by dividing the number of enterospheres and enteroids present at day 4 or 8 by the number of enterospheres present on day 1 in each well. This provides a measurement of how many enterospheres initially plated were able to survive and grow in crypt culture conditions. Efficiency measurements revealed a trend for decreased enteroid survival in old *vs.* young at day 4 post plating and a significant decrease in enteroid survival in old animals at day 8 post plating (Figure 1B). Crypt-derived enteroids typically begin to show bud structures by 3-7 days post plating [31]. Each bud represents a crypt structure that containing stem and progenitor cells and the number of buds provide a surrogate for IESC function [32]. The numbers of buds per enteroid were counted at days 4 and 8 post plating, categorizing enteroids with 2 buds or fewer as less complex, and enteroids containing 3 or more buds as more complex. Following 4 days in culture there was no difference in the enteroid complexity between young and old (Figure 1C). By day 8 post plating, enteroids from old mice showed a decrease in complexity compared to those from young mice as significantly fewer enteroids from old animals contained 3 or more buds (Figure 1C). At 15 days post plating very complex enteroids had formed from the crypts derived from young mice, while the enteroids formed from old mice were much less complex (Figure 1A).

### Increased villus height and Paneth cell number in small intestine of old mice

**Figure 2:**
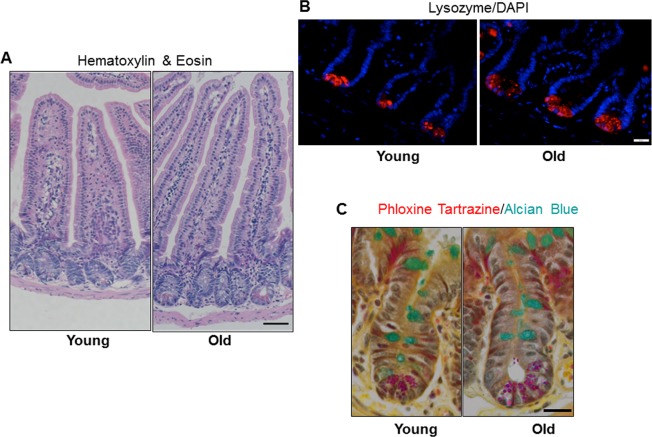
Increased jejunal villus height and number of Paneth cells per crypt with age (**A**) Representative hematoxylin and eosin stained sections of jejunal cross-sections from young and old mice showing crypt and villus architecture. Images were taken at 10x magnification. Scale bar, 50μm. (**B**) Representative images of crypt sections stained for the Paneth cell marker Lysozyme and nuclear marker DAPI. Magnification: 40x, Scale bar : 20μm. n=6 animals per group. (**C**) Representative images of crypt sections stained for the Phloxine Tartrazine and the Goblet cell marker Alcian Blue. Magnification: 40x, Scale bar : 20μm. n=5 young and 6 old animals.

Jejunal morphology and morphometry and the presence of differentiated cell types were assessed by histology. Results revealed no significant difference in crypt depth, crypt or villus density, total number of cells per crypt, or mucosal circumference between young and old mice, but demonstrated a significant increase in villus height in old *vs*. young mice (Table 1 and Figure 2A). Immunofluorescence for lysozyme, a Paneth cell bio-marker, revealed a small but significant increase in the number of Paneth cells per crypt in old *vs.* young (Table 1 and Figure 2B). Alcian Blue positive goblet cells were quantified and revealed no change in the number of mucus producing goblet cells between the young and old mice (Table 1 and Figure 2C).

**Table 1:** Morphometric data and numbers of Paneth cells or goblet cells in the jejunum in young and old mice

Measure	Young	Old
	Mean (SEM)	Mean (SEM)
Crypt Depth (μm)	50.7 (1.8)	50.6 (2.5)
Total # epithelial cells per crypt	34 (3)	30 (1)
Villus Height (μm)	**263.2 (26.1)**	361.7* (30.0)
Crypt Density (crypts/cm)	145 (18)	159 (19)
Villus Density (villi/cm)	40 (5)	42 (3)
Circumference (cm)	0.97 (0.06)	0.96 (0.05)
# Lysozyme+ Paneth cells/crypt	**3.6 (0.2)**	4.3* (0.1)
# Alcian Blue+ Goblet cells/crypt	5.5 (0.7)	5.5 (0.6)

n=6 animals per group for all measurements, *p<0.05 Young vs Old, unpaired t test.

### Old mice exhibit increased IESC number and enhanced IESC proliferation

**Figure 3:**
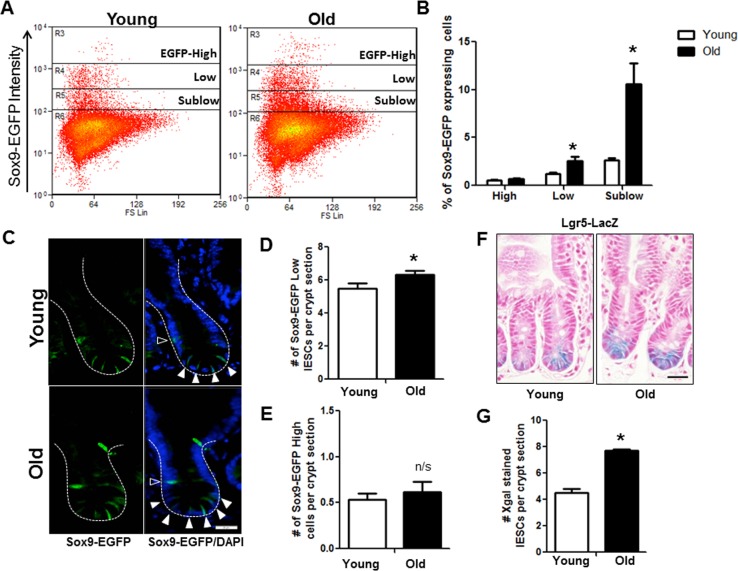
Increased IESC in old mice (**A**) Representative flow cytometry data of Sox9-EGFP expressing cells in young and old Sox9-EGFP reporter mice. Gate: R3=Sox9-EGFP^High^, R4=Sox9-EGFP^Low^, R5=Sox9-EGFP^Sublow^. (**B**) Relative abundance of different Sox9-EGFP expressing cells measured by flow cytometry. n=19 animals per group, *p<0.05 Young vs. Old, unpaired t test. (**C**) Representative images of crypt sections from young and old Sox9-EGFP reporter mice stained with EGFP and the nuclear marker DAPI. Sox9-EGFP^Low^ IESC marked by closed triangles. Sox9-EGFP^High^ EEC marked by open triangles. Magnification : 40x, Scale bar : 20μm. (**D**) Quantification of the number of Sox9-EGFP^Low^ IESC counted per crypt section. n=8 young and 9 old animals, *p<0.05 Young vs. Old, unpaired t test. (**E**) Quantification of the number of Sox9-EGFP^High^ cells counted per crypt section. n=6 young and 7 old animals. (**F**) Representative images of Xgal stained crypt sections from young and old Lgr5-LacZ reporter mice. Magnification : 40x, Scale bar : 20μm. (**G**) Quantification of the number of Xgal stained Lgr5-LacZ IESC counted per crypt section. n=4, p<0.05 Young vs. Old, unpaired t test.

Flow cytometry on IEC isolated from Sox9-EGFP mice revealed significantly more IESC (Sox9-EGFP^Low^) and progenitor cells (Sox9-EGFP^Sublow^) in old *vs*.to young (Figure 3A and 3B). However, the Sox9-EGFP^High^ cell population that contains enteroendocrine cells (EECs) and a reserve stem cell population activatable upon injury and regeneration [25] did not differ between young and old mice (Figure 3A and 3B). Results were confirmed by EGFP immunofluorescence, which revealed no change in the number of Sox9-EGFP^High^ cells per crypt in old *vs.* young (Figure 3C and 3E) but demonstrated a significant increase in the number of Sox9-EGFP^Low^ IESC per crypt in old animals (Figure 3C and 3D). This was further confirmed in the Lgr5-LacZ IESC reporter mouse model [23] where expansion of Lgr5-LacZ IESC was observed in old *vs.* young (Figure 3F and 3G). Of note, using histology it is not possible to reliably quantify Sox9-EGFP^Sublow^ progenitor cells by EGFP immunofluorescence because the very low EGFP expression cannot be readily distinguished from background.

**Figure 4:**
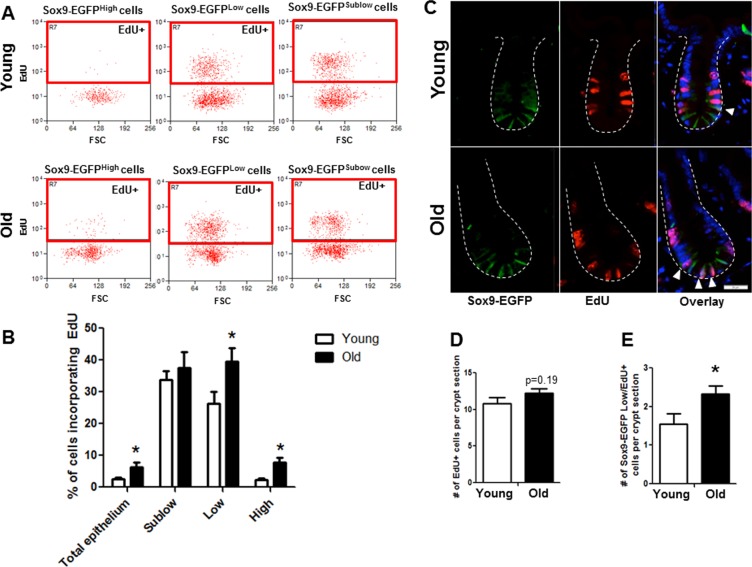
Old mice exhibit increased proportion of Sox9-EGFP^Low^ IESC and Sox9-EGFP^High^ cells in S-phase as assessed by flow cytometry and histology (**A** and **B**) Relative abundance of Sox9-EGFP expressing populations that have incorporated EdU was measured by flow cytometry. n=6 animals per group, *p<0.05 Young vs. Old, unpaired t test. (**C**) Representative images of crypt sections from young and old Sox9-EGFP mice stained with EGFP, S-phase marker EdU and nuclear marker DAPI. Closed arrows indicate Sox9-EGFP^Low^ IESC in S-phase. (**D**) Number of total crypt cells incorporating EdU per Sox9-EGFP crypt section. n=6 animals per group. (**E**) Number of Sox9-EGFP^Low^ IESC incorporating EdU per crypt section. n=6 animals per group, *p<0.05 Young vs. Old, unpaired t test.

Flow cytometry of dissociated jejunal IEC from young and old Sox9-EGFP reporter mice detected incorporation of the S-phase marker EdU in 3 distinct EGFP-expressing cell populations (Figure 4B). As expected, rapidly dividing Sox9-EGFP^Sublow^ progenitor cell population showed the most EdU positive cells compared to other Sox9-EGFP populations (Figure 4).

Based on percentage of cells incorporating EdU, there was no detectable difference in proliferation of Sox9-EGFP^Sublow^ progenitors between young (33.7±2.7%) and old (37.6±5.0%) (Figure 4). How-ever, there was a significant increase in the percentage of EdU-positive Sox9-EGFP^Low^ IESC in old (39.5±4.3%) *vs.* young (26.3±3.8%), indicating hyperproliferation of IESC in old mice. The percentage of EdU-labeled Sox9-EGFP^High^ cells was significantly higher in old (7.8±1.6%) *vs.* young (2.4±0.5%) (Figure 4). This provides evidence that old mice have a greater proportion of “activatable reserve” IESC in S phase.

**Figure 5:**
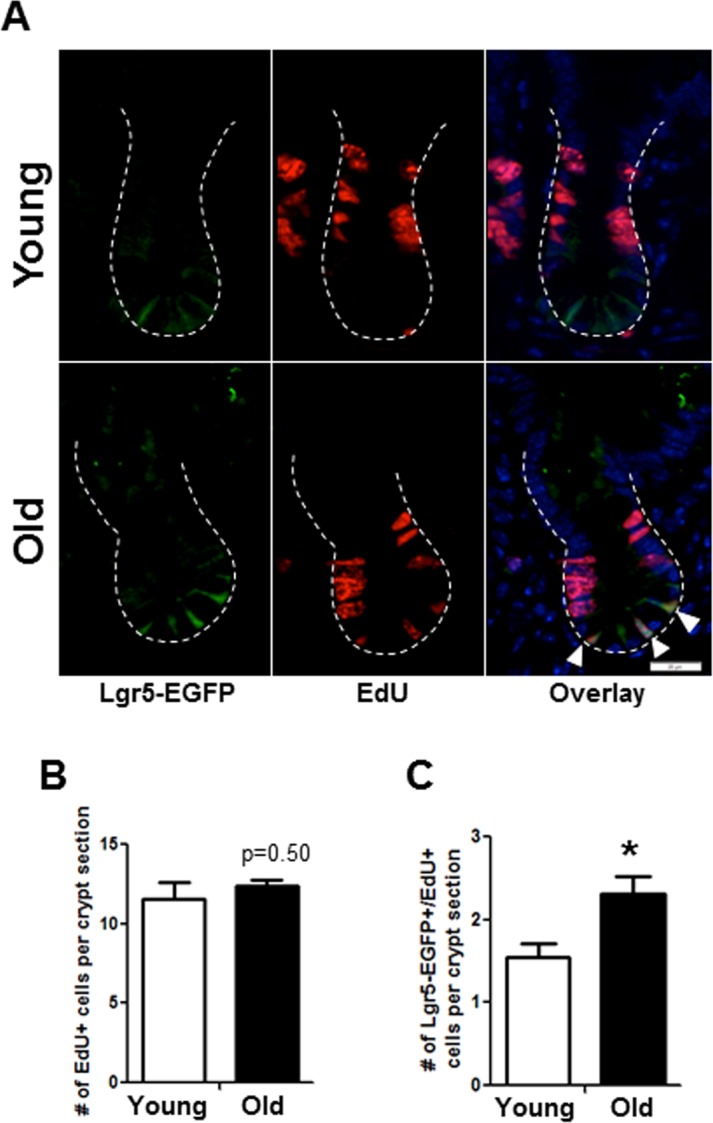
Histology to demonstrate increased proportion of Lgr5-EGFP IESC in S-phase in old mice (**A**) Representative images of crypt sections from young and old Lgr5-EGFP mice stained with EGFP, S-phase marker EdU and nuclear marker DAPI. Closed arrows indicated Lgr5-EGFP IESC in S-phase. (**B**) Number of total crypt cells incorporating EdU per crypt section. n=5 young and 6 old animals. (**C**). Number of Lgr5-EGFP IESC incorporating EdU per crypt section. n=5 young and 6 old animals, *p<0.05 Young vs. Old, unpaired t test. Magnification: 40x, Scale bar : 20μm for all images

Immunofluorescence for EdU revealed no difference in the total number of EdU positive cells per crypt in old *vs.* young (Figure 4C, 4D). However, co-immunofluores-cence for EdU and GFP revealed a significant increase in the number of EdU positive Sox9-EGFP^Low^ IESC located at the crypt base in old mice (Figure 4C, 4D). Results were confirmed in an independent IESC reporter mouse model, Lgr5-EGFP [23] (Figure 5A-C), which revealed significantly more Lgr5-EGFP IESC co-labeled with EdU in old (2.3±0.2) *vs.* young (1.5±0.2) (Figure 5C). Together these data provide strong evidence that aging is associated with a selective increase in proliferation and/or altered cell cycle dynamics in IESC of old mice.

### Old mice exhibit increased apoptosis of the small intestinal epithelial cells

**Figure 6:**
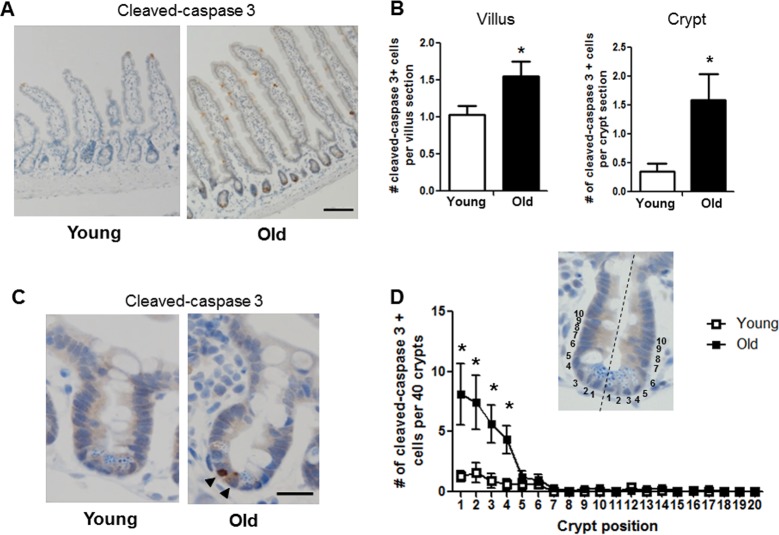
Increased proportion of small intestinal epithelial cell apoptosis with age (**A**) Representative image of crypt and villus sections stained for cleaved caspase-3. Magnification : 10x, Scale bar : 100μm. (**B**) Quantification of the number of cleaved caspase-3 positive cells per villus section (n=7 animals per group) and per crypt section (n=10 young and 9 old animals). *p<0.05 Young vs. Old, unpaired t test. (**C**) Representative image of crypt sections stained for cleaved caspase-3. Magnification: 40x, Scale bar: 20μm. (**D**) Quantification of the location of cleaved caspase-3 positive cells per 40 crypts per animal by position. Inlay shows method of identifying cell position within crypt. n=10 young and 9 old animals, *p<0.05 Young vs. Old, unpaired t test.

Given the age-associated IESC dysfunction indicated by our results in the *ex vivo* enteroid culture system, we tested whether apoptosis was increased in the small intestinal crypt of old *vs*. young mice. Cleaved caspase-3 immunohistochemistry revealed a significant increase in the number of apoptotic cells per crypt section in old *vs.* young (Figure 6A, 6B-right and 6C). The location of cleaved caspase-3 positive cells within the crypt was assessed by dividing the crypt in half lengthwise and counting the position of apoptotic cells from the base of the crypt (Figure 6D). Most apoptotic cells in old animals were at the base of the crypt in positions 1-4 (Figure 6C and 6D), indicating preferential increases in apoptosis within the IESC zone. Old mice showed a significant increase in cleaved caspase-3 positive cells in the villi (Figure 6A and 6B-left), that were strikingly located along the villus epithelium rather than the villus tip.

### Aging-associated changes in gene expression

**Figure 7:**
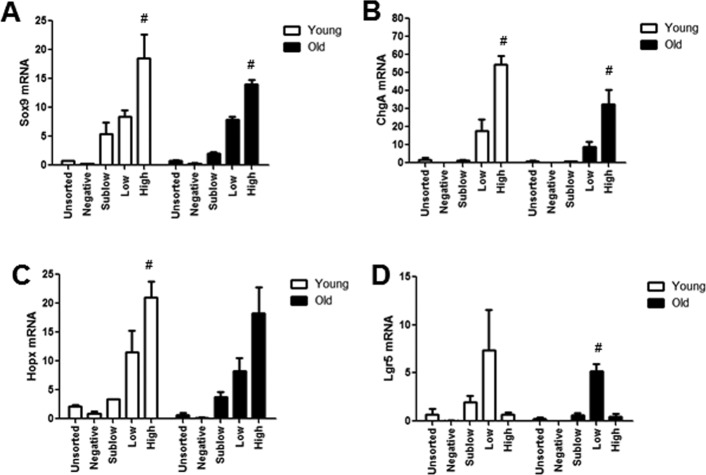
Isolated Sox9-EGFP cell populations from young and old mice are enriched for known biomarkers associated with specific population Sox9-EGFP cell populations isolated using FACS underwent high throughput qRT-PCR for the genes (**A**) Sox9, (**B**) ChgA, (**C**) Lgr5, (**D**) Hopx. n≥3 animals per group, #p<0.05 compared to all other Sox9-EGFP populations, 1-way ANOVA, Bonferroni. n=4 per group.

To gain insight into molecular mechanisms or biomarkers of age associated functional changes in IESC and other cell populations between old and young mice, we used FACS (Fluorescence Activated Cell Sorting) coupled with Fluidigm mRNA profiling. First we validated that profiles of Sox9 mRNA and biomarkers of specific IEC subtypes exhibited predicted profiles in isolated cell populations from young and old Sox9-EGFP mice (Figure 7A-D). Our data verified that the expression profiles of Sox9 mRNA in FACS-isolated cells matched the profiles of Sox9-EGFP used to FACS isolate cell populations (Figure 7A). Furthermore, Sox9 mRNA profiles across Sox9 negative, sublow, low and high populations were consistent between young and old. The IESC specific marker *Lgr5* was enriched in the Sox9-EGFP^Low^ population from both young and old, consistent with phenotypic similarities between Sox9-EGFP^Low^ and Lgr5-EGFP-expressing IESC (Figure 7B). Sox9-EGFP^High^ populations from young and old were enriched for EEC specific mRNA *ChgA* (Figure 7C) and *Hopx*, a putative biomarker of activatable reserve IESC (Figure 7D). To gain insight into the mechanisms driving aging-associated IESC expansion and hyper-proliferation, we examined expression of mRNAs involved in cell cycle, apoptosis and maintenance of homeostasis in cell populations isolated from Sox9-EGFP mice. Consistent with histology and flow cytometry data suggesting increased proportion of active Lgr5-expressing and activatable reserve IESC in S-phase in old mice, we observed a significant increase in *Ccnd1* and *Cdk6* mRNAs in Sox9-EGFP^Low^ cells and a dramatic increase in *Ccnd1 mRNA* in Sox9-EGFP^High^ cells from old *vs.* young (Table 2A, 2B). *Ccnd1* and *Cdk6* are required for cell cycle progression and activate G1 to S phase transition [33, 34]. Additional mRNAs involved in regulating the cell cycle and proliferation were significantly different in cell populations from old *vs*. young including *Max* in Sox9-EGFP^High^ and *p21* in Sox9-EGFP^Low^ (*p21*) (Table 2A, 2B). Consistent with increased levels of apoptosis observed based on localization of cleaved caspase-3 in the crypt base of old animals, Fluidigm data revealed increased levels of mRNAs encoding *p53* and *Perp* specifically in Sox9-EGFP^Low^ IESC from old mice (Table 2B). We found a significant increase in the mRNA encoding *Nrf2*, a marker of oxidative stress, in Sox9-EGFP^Low^ IESC from old mice (Table 2B). Significantly decreased levels of mRNAs associated with proliferation, apoptosis and homeostasis were observed in the old Sox9-EGFP^Sublow^ progenitor cell population, including *Pten*, *Yap1*, *Ephb2*, *Notch2* and *Bbc3* (Table 2C). H2afx mRNA levels were increased specifically in Sox9-EGFP^Negative^ cells from old mice, consistent with and providing a potential biomarker of age-associated increases in apoptosis of differentiated cells on the villus. Decreased levels of mRNAs known to be associated with tissue homeostasis with aging in Sox9-EGFP^Negative^ cells from old mice are consistent with increases in villus height, a sign of altered homeostasis.

**Table 2:** mRNAs significantly different in specific cell populations isolated from old versus young Sox9-EGFP mice

**A. Sox9-EGFP High Gene Regulation**			
**mRNAs upregulated specifically in Sox9-EGFP High cells**			
**Symbol**	**Gene name**	**Fold Change**	**Role**
*Ccnd1*	cyclin D1	>10	cell cycle progression [33]
*Max*	MYC associated factor X	1.97	regulates proliferation [57]
**mRNAs downregulated specifically in Sox9-EGFP High cells**			
**Symbol**	**Gene name**	**Fold Change**	**Role**
*Irs1*	insulin receptor substrate 1	−2.09	deficiency induces ISC apoptosis [58]
**B. Sox9-EGFP Low Gene Regulation**			
**mRNAs upregulated specifically in Sox9-EGFP Low cells**			
**Symbol**	**Gene name**	**Fold Change**	**Role**
*p53*	tumor protein 53	>10	induces apoptosis [59]
*Nrf2*	nuclear factor, erythroid derived 2, like 2	2.62	protects against oxidative stress [60]
*Cdk6*	cyclin-dependent kinase 6	2.43	cell cycle progression [34]
*Ccnd1*	cyclin D1	2.3	cell cycle progression [33]
*Perp*	TP53 apoptosis effector	1.94	induces apoptosis [52]
**mRNAs downregulated specifically in Sox9-EGFP Low cells**			
**Symbol**	**Gene name**	**Fold Change**	**Role**
*Cdkn1A/p21*	cyclin-dependent kinase inhibitor 1A	−2.16	inhibits proliferation [61]
**C. Sox9-EGFP Sublow Gene Regulation**			
**mRNAs downregulated specifically in Sox9-EGFP Sublow cells**			
**Symbol**	**Gene name**	**Fold Change**	**Role**
*Pten*	phosphatase and tensin homolog	−1.26	regulates ISC proliferation [62]
*Yap1*	yes-associated protein 1	−1.81	regulates proliferation and homeostasis [63]
*Ephb2*	EPH receotpr B2	−2.19	promotes proliferation and controls migration [64]
*Notch2*	notch 2	−2.68	regulates intestinal homeostasis and differentiation [65]
*Bbc3/Puma*	BCL2 binding component 3	−3.31	deficiency blocks apoptosis [66]
**D. Sox9-EGFP Negative Gene Regulation**			
**mRNAs upregulated specifically in Sox9-EGFP Negative cells**			
**Symbol**	**Gene name**	**Fold Change**	**Role**
*H2afx*	H2A histone family, member X	3.4	DNA damage repair [67]
**mRNAs downregulated specifically in Sox9-EGFP Negative cells**			
**Symbol**	**Gene name**	**Fold Change**	**Role**
*Ephb2*	EPH receotpr B2	−42.5	promotes proliferation and controls migration [64]

Sox9-EGFP cell populations from young and old mice were isolated by FACS and mRNA levels were assessed by high throughput qRT-PCR using Fluidigm. The table shows mRNAs that were significantly up- or down–regulated, in different cell populations isolated from old versus young Sox9-EGFP mice, and the fold change in old versus young mice. A. Sox9-EGFP^High^ activatable reserve IESC/enteroendocrine cell populations; B. Sox9-EGFP^Low^ IESC; C. Sox9-EGFP^Sublow^ progenitor cells and D. Sox9-EGFP^Negative^ progenitor cells. In each cell population, values normalized to a pooled unsorted sample were compared for statistical difference between Young vs Old cells; n≥3 animals per group; p<0.05 was considered significant. The Tables show the fold difference for statistically different mRNAs in specific cell populations from old vs. young mice.

## DISCUSSION

Until 2007 the study of IESC and the effect of aging on IESC was hampered by a lack of specific biomarkers. Our findings in the Sox9-EGFP reporter mouse, confirmed in Lgr5-EGFP and Lgr5-LacZ reporter mice models, provide new and direct evidence that aging is associated with increases in IESC number, proliferation, and increases in IESC apoptosis in vivo and functional impairment of isolated IESC in vitro. Our findings of increased villus height and expansion of Paneth cells in old mice indicate altered intestinal homeostasis with aging.

Consistent with our findings, aging-associated changes in small intestinal morphology including increased in villus height have been reported in mice [35], rats [36] and humans [37]. Previous findings that old rats exhibit decreased absorption of fatty acids and glucose indicate aging-associated functional impairment in nutrient absorption [38]. Aging-associated increases in villus height may therefore represent a compensatory mechanism to reduce risk of malnutrition with aging. Our results showing increased cleaved caspase-3 positive cells on villi of old mice provide additional support for aging-associated functional impairment of differentiated enterocytes. Our findings also support a model where increased villus height in old animals is maintained by an expanded pool of hyperproliferative IESC that is needed to generate additional differentiated enterocytes to enhance nutrient absorption.

Paneth cells release trophic factors in the IESC niche, contribute to maintained IESC function [39], and secrete antimicrobial peptides to provide host defense against microbes [40]. Age-associated increases in bacterial load have been reported in the intestinal lumen of *Drosophila* and were associated with increased IESC proliferation [41]. Furthermore, previous studies in mice and humans reported changes in intestinal microbiome with age including changes in proportion of major microbes and decreased overall microbiota diversity [42, 43]. Our observed increases in Paneth cell number in old mice therefore suggest an adaptive response that may promote IESC expansion and could reflect age-associated changes in microbiota.

Our findings in the Sox9-EGFP mouse model and confirmative data in Lgr5-EGFP and Lgr5-LacZ IESC provide, to our knowledge, the first direct evidence for age-associated expansion and hyperproliferation of mammalian IESC. Our findings that crypts from old animals have reduced enteroid forming ability and yield enteroids with reduced complexity compared with ente-roids formed from young crypts provide new evidence for age-associated functional impairment of IESC.

The Sox9-EGFP mouse model offers the opportunity to study and isolate progenitor cells (Sox9-EGFP^Sublow^) and Sox9-EGFP^High^ cells that contain EEC cells and an activatable reserve stem cell population [25]. A recent study suggested that subsets of secretory EEC cells can act as reserve stem cells in humans [45]. Interestingly, we observed an increased proportion of Sox9-EGFP^High^ cells in S phase of the cell cycle in old mice, yet no expansion in cell number. This provides novel evidence that aging is associated with activation and proliferation of reserve stem cells as a mechanism to maintain epithelial renewal during aging.

Based on *in vivo* findings of aging-associated IESC hyperproliferation and increased apoptosis in the stem cell zone of the crypt, we assessed levels of mRNAs encoding proteins linked to cell cycle regulation, oxidative stress, DNA damage and apoptosis in the different Sox9-EGFP cell populations isolated from young and old mice (Probes for mRNAs showing significant differences between cells from young and old mice are described in Supplementary Table 1). Sox9-EGFP^High^ cells and Sox9-EGFP^Low^ IESC from old mice exhibited increased levels of mRNAs encoding proteins that accelerate G1 to S phase transition including *Ccnd1*, *Cdk6*, *Max* and *Mlx* [46]. IESC hyper-proliferation may increase the probability of genomic instability, triggering a DNA damage response and apoptosis [46]. Elevation of *Nrf2* mRNA in Sox9-EGFP^Low^ IESC from old mice adds support to a model of aging-associated cellular and molecular damage in IESC [48, 49]. *Nrf2* promotes accumulation of reactive oxygen species (ROS), which causes DNA damage, and has been linked to aging in both drosophila and mammals [47]. Elevation of mRNAs encoding proteins known to stimulate cell apoptosis including *p53*, *Sirt7*, *Max*, *Bak1* and *Bax* in actively cycling Sox9-EGFP^Low^ IESC provide new evidence for IESC-specific mediators linked to IESC dysfunction. p53 is induced in response to DNA damage or cellular stress and activates cell cycle arrest, apoptosis or senescence pathways [50, 51]. Our observed induction of both *p53* mRNA and the downstream mediator, *Perp* [52, 53] in old IESC support a model of aging-linked oxidative stress and DNA damage-induced apoptosis. Overall, mRNA profiling data on young and old IESC and differentiated lineages provide new evidence that aging is associated with altered expression of multiple genes involved in regulation of cell cycle, proliferation, apoptosis, DNA damage and repair.

**Figure 8:**
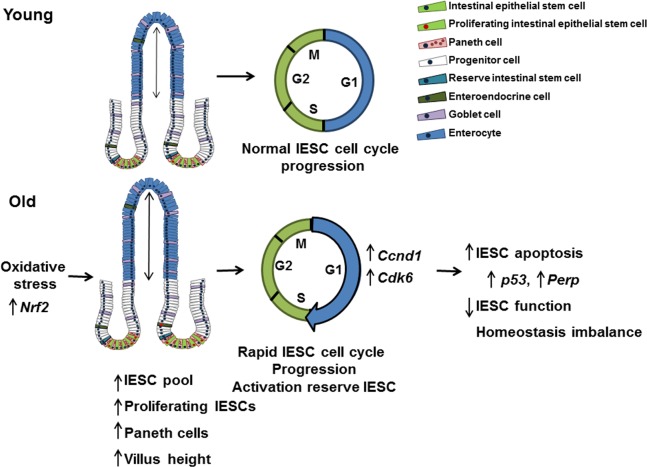
Model outlining the effects of aging on the small intestine This model suggests that IESC specific changes in proliferation and cell cycle regulation with age are the result of oxidative stress. Cell cycle is accelerated in IESC resulting in IESC hyperproliferation and an increased IESC pool. The observed increase in Paneth cells may be required to support the increased number of IESC per crypt in aged animals. Increased IESC proliferation with age may lead to, increased DNA damage, resulting in p53 activated IESC apoptosis and decreased IESC function.

Together our findings support a model where dysregulated cell cycle progression and evasion of cell cycle checkpoints promotes age-related hyper-proliferation and expansion of actively cycling IESC, increasing cellular stress, DNA damage, and leading to increased apoptosis and impaired IESC function (Figure 8). This model is consistent with aging-associated dysfunction and loss of stem cell populations in other organs [54]. Observed increases in proliferation of Sox9-EGFP^High^ cells, indicate that reserve, activatable IESC are mobilized to compensate for hyper-proliferation but impaired function of actively cycling IESC. Age-associated IESC hyper-proliferation may be necessary to support increased villus height, compensate for increased villus apoptosis and maintain functions of the intestinal epithelium with aging. Our identification of mediators specifically altered in IESC from old mice provides a basis for translational studies of mediators and pathways of IESC aging in humans and to identify potential targets for new approaches or therapies that may ameliorate aging-associated dysfunction or disease of the intestine. Importantly, our IESC-reporter mice provide the models for preclinical studies of the effect of potential interventions specifically on IESC aging.

## MATERIALS AND METHODS

### Mouse models

Sox9-EGFP mice on an outbred CD-1 background were maintained as heterozygotes and genotyped as previously described [24, 28]. Lgr5-EGFP-IRES-CreERT2 (Lgr5-EGFP) mice on a C57BL/6 background were ordered from The Jackson Laboratory (Bar Harbor, ME) and maintained as heterozygotes. Original Lgr5-LacZ mice on a mixed background were generously provided by Lexicon Pharmaceuticals (The Woodlands, TX). Lgr5-LacZ mice on C57BL/6 back-ground that were used in this study were generated after 10 generations of backcrossing the original Lgr5-LacZ mice with C57BL/6 wild type mice. Young mice studied were 2 to 4 months old. Old mice were 18 to 24 months old. Young and old female and male mice were studied. For the old study groups, females were 20-24 months old and males 18-20 months old. These ages were selected based on pilot studies demonstrating no overt signs of disease in female or male mice at these ages.

### Crypt isolation and culture

Jejunal crypts were isolated and in vitro crypt culture was performed as previously described [32]. Approximately 30 crypts were plated into 24 well plates in 10μl Low Growth Factor Matrigel (BD Biosciences, Franklin Lakes, NJ) containing 50ng/ml EGF (R&D Systems, Minneapolis, MN), 100ng/ml Noggin (Peprotech, Rocky Hill, NJ) 1μg/ml R-spondin (R&D Systems, Minneapolis, MN) and 10uM Y-27632 (Sigma-Aldrich, St. Louis, MO). After polymerization 250μl Advanced DMEM-F12 containing N2 supplement (Invitrogen, Carlsbad, CA), B27 supplement without vitamin A (Invitrogen, Carlsbad, CA), 10mM HEPES (Invitrogen, Carlsbad, CA), Glutamax (Gibco-Invitrogen, Carlsbad, CA) and Pen/Strep (Gibco-Invitrogen, Carlsbad, CA) was added to each well. Growth factors were added every 2 days at the same concentrations as the initial plating except R-spondin, which was reduced to 500ng/ml. Medium was changed every 4 days. The number of enterospheres or enteroids and their complexity was recorded at days 1, 4 and 8-post plating. Representative images of cultures were taken using an inverted fluorescent microscope (Olympus IX83).

### Tissue harvest

Sox9-EGFP, Lgr5-EGFP and Lgr5-LacZ mice were euthanized with a lethal dose of Nembutal (150μg/g body weight). To identify cells in S-phase, 5-ethynyl-2′ deoxyuridine (EdU; Sigma-Aldrich, St. Louis, MO) was administered by intraperitoneal injection at a dose of 100μg/25 g of body weight 90 minutes prior to Nembutal injection. The entire intestine was then removed and flushed with cold PBS. The jejunum was dissected on ice for further analyses.

### Immunostaining

Intestinal segments were splayed open and fixed overnight in freshly made 4% paraformaldehyde at 4°C, washed, incubated in 10% sucrose in PBS at 4°C overnight and incubated in 30% sucrose in PBS at 4°C until embedding. Tissues were embedded in Optimal Cutting Temperature compound (OCT) and sectioned at 5-7-μm thickness. Antigens were retrieved in 10mM sodium citrate buffer (pH=6) followed by blocking with 10% normal goat serum. After blocking, samples were incubated overnight at 4°C with each of the following primary antibodies: GFP (1:500, chicken; Aves Labs, Tigard, OR), lysozyme (1:500, rabbit; Leica Biosystems, Buffalo Grove, IL), cleaved caspase-3 (1:500, rabbit; Abcam, Cambridge, UK). For immunofluorescence, slides were washed and incubated with the following secondary antibodies: goat-anti-chicken-AlexaFluor 488 (1:400; Invitrogen, Carlsbad, CA) or goat-anti-rabbit-Cy3 (1:400; Jackson Immuno-Research Laboratories, West Grove, PA). Slides were then mounted using DAPI containing mounting medium (Electron Microscopy Sciences, Hatfield, PA) to visualize nuclei. Peroxidase with Vectastain avidin-biotin complex (ABC kit; Vector Laboratories, Burlingame, CA) was used for immune peroxidase based staining. Morphometric analyses were performed on formalin fixed, paraffin embedded tissue cross-sections. Tissues were washed well and slides (5-7 μm) were stained with hematoxylin and eosin or Phloxine Tartrazine and Alcian Blue. Fluorescent images were captured using an inverted fluorescence microscope (IX83; Olympus, Center Valley, PA). White light images were captured using an upright light microscope (AX10; Zeiss, Oberkochen, Germany). At least 20 crypts were counted per animal and number of animals per group is indicated in figure legends.

### Phloxine Tartrazine and Alcian blue staining

Alcian Blue Phloxine Tartrazine staining was performed based on previous reports [55]. Briefly, slides were stained with Alcian blue (Sigma-Aldrich, St. Louis, MO) for 30 minutes, followed by alum hematoxylin (BioCare, Concord, CA) for 20 seconds, staining with 0.5% phloxine in calcium chloride (Fisher Scientific, Waltham, MA) for 20 minutes, and finally incubate in a saturated solution of tartrazine in Cellosolve (Fisher Scientific, Waltham, MA) for 3-5 minutes. Stained sections were observed using light microscopy.

### β-Galactosidase staining

Jejunal cross sections from Lgr5-LacZ mice were dissected, washed with PBS, then fixed at 4°C using 2% paraformaldehyde (PFA)/0.2% glutaraldehyde in PBS for 2 hours. Tissues were then rinsed for 20 minutes at room temperature in 0.1M Phosphate Buffer, pH7.3 and permeabilized by incubation in 0.1M Phosphate Buffer, pH7.3 containing 0.1% Triton-X 100. Tissues were then transferred to X-gal solution (1g/liter X-Gal) in 0.1M Phosphate Buffer, pH7.3 with 5 mmol/liter of potassium ferrocyanide, 5 mmol/liter of potassium ferricyanide and 0.1% Triton X-100) overnight in the dark at room temperature. Tissues were then washed twice with PBS and post-fixed in 4% paraformaldehyde at 4°C overnight. Subsequently, tissue was washed, dehydrated in 70% ethanol and embedded in paraffin. 5-7 μm sections were counterstained with nuclear fast red and observed using light microscopy. At least 40 crypts were counted per animal and number of animals per group is indicated in figure legends.

### Intestinal epithelial cell isolation, flow cytometry and FACS

Intestinal epithelial cells were isolated from the jejunum of young and old Sox9-EGFP mice for flow cytometry as previously described [25]. Dissociated intestinal epithelium was stained with propidium iodide solution (Sigma-Aldrich, St. Louis, MO) to exclude dead cells. To assess the proportion of Sox9-EGFP cells in S-phase by flow cytometry, an equal number of dissociated intestinal epithelial cells was fixed and stained for EdU using the Click-it EdU AlexaFluor 594 Kit and following the manufacturer's instructions (Invitrogen, Carlsbad, CA). Gates were placed based on levels of Sox9-EGFP expression and back gating was performed to assess the percentage of cells incorporating EdU in each Sox9-EGFP expressing cell population. Quantification of different Sox9-EGFP expressing populations was performed using a Cyan flow cytometer and Summit software v4.3 (Beckman Coulter, Brea CA). FACS on Sox9-EGFP cells for population analysis was performed using the MoFlow XDP cell sorter (Beckman Coulter, Brea, CA). FACS on Sox9-EGFP cells for single cell analysis was performed using the SH800Z cell sorter (Sony Biotechnology Inc., San Jose, CA). Immune cells were excluded from the sort by gating out CD31+ (BioLegend, San Diego, CA) and CD45+ (BioLegend, San Diego, CA) cells. Dead/dying cells were excluded from the sort by gating out SytoxBlue+ (ThermoFisher Scientific, Waltham, MA) and Annexin V+ (Invitrogen, Carlsbad, CA) cells. Cells were sorted into Sorting Medium composed of Advanced DMEM/F12, 100x N2 Supplement, 50x B27 Supplement, 1mM HEPES), 100x Penicillin/Streptomycin, 100x Glutamax all from (Gibco-Invitrogen, Carlsbad, CA) and supplemented with 10uM Y27632 (Invitrogen, Carlsbad, CA), and 1mM N-Acetylcysteine (NAC; Sigma-Aldrich, St. Louis, MO).

### High throughput cell population qRT-PCR using Fluidigm

Total RNA was isolated from FACS sorted Sox9-EGFP populations using the RNAqueous-Micro Total RNA Isolation Kit (Ambion-ThermoFisher Scientific, Waltham, MA) according to the manufacturer's instructions. RNA was quantified by measuring absorption at 260 nm using a Nanodrop photometer (Tecan, Morrisville, NC) and underwent DNase treatment to remove genomic DNA. Reverse transcription was performed on 25 μg RNA using Cells Direct One-Step qRT-PCR Kit (Invitrogen, Carlsbad, CA). Specific target amplification (STA) of 96 genes was performed on cDNA from the different FACS sorted populations isolated from Sox9-EGFP mice using Taqman primers purchased from Applied Biosystems. These target genes included population specific markers, genes that stimulate cell proliferation, apoptosis, DNA damage and maintain homeostasis. Preparation and loading of Fluidigm 2 × 48.48 Dynamic Array integrated fluidic circuit (IFC) was performed according to the manufacturer's instructions (Fluidigm, South San Francisco, CA). Briefly, the chip was primed, loaded with samples and primer reaction mixes and run on the BioMark™ HD System. Data analysis was accomplished with the Fluidigm Real-Time PCR Analysis software. Resulting cycle threshold (Ct) values were analyzed by calculating the relative gene expression (fold change) using the 2^−ΔΔCt^ method [56]. β-actin was used as the reference gene to normalize mRNA input and allow accurate comparisons of results between samples. An unsorted, pooled RNA sample was set as the control sample to which all other samples were compared. GraphPad Prism 5 was used to perform a 1-way ANOVA with Bonferroni post-hoc test to test for differences in levels of mRNA across different cell populations and between cells from young and old mice.

### Statistical analysis

Data are expressed as mean ± SEM. For histology morphometry, histology, flow cytometry and *in vitro* culture experiments, unpaired Student's *t*-tests were performed to compare data from young and old mice. For high throughput qRT-PCR experiments one-way ANOVA with Bonferroni post hoc test was performed for comparisons of multiple groups. A P value of less than 0.05 was considered statistically significant.

## SUPPLEMENTARY MATERIAL TABLE


