# Supplementary material for: Aging effects on intestinal homeostasis associated with expansion and dysfunction of intestinal epithelial stem cells
Source: Aging (Albany NY). 2017 Aug 29;9(8):1898–915. doi: 10.18632/aging.101279 (PMC5611984; doi:10.18632/aging.101279)
Supplement: Supplementary file 1 [file aging-09-1898-s001.pdf]

SUPPLEMENTARY MATERIAL

Supplementary Table 1. TaqMan probes used in high throughput Fluidigm-based qRT-PCR analysis of IESC populations.

Gene	TaqMan ID	Pathway
Bbc3	Mm00519268_m1	Apoptosis
Ccnd1	Mm00432359_m1	Cell Cycle
Cdk6	Mm00432051_m1	Cell Cycle
Ephb2	Mm01181021_m1	Cell Cycle
H2afx	Mm00515990_s1	Oxidative Stress
Irs1	Mm01278327_m1	Apoptosis
Max	Mm00484802_g1	Cell Cycle
Nrf2	Mm00477784_m1	Oxidative Stress
Notch2	Mm00803077_m1	Cell Cycle
p21	Mm00432448_m1	Cell Cycle
p53	Mm01731290_g1	DNA Damage and Apoptosis
Perp	Mm00480750_m1	Apoptosis
Pten	Mm00477208_m1	Cell Cycle
Yap1	Mm01143263_m1	Cell Cycle
